# The androgen receptor plays different roles in macrophage-induced proliferation in prostate stromal cells between transitional and peripheral zones of benign prostatic hypertrophy

**DOI:** 10.17179/excli2017-335

**Published:** 2017-06-22

**Authors:** Dongliang Xu, Xingjie Wang, Chenyi Jiang, Yuan Ruan, Shujie Xia, Xiaohai Wang

**Affiliations:** 1 Department of Urology, Shanghai General Hospital, Address: No. 100 Haining Road, Hongkou district, Post code: 200080, Shanghai, China; Telephone: +86 13916482122 (Wang); Telephone: +86 15301655577 (Xia)

**Keywords:** stromal cell, macrophage, androgen receptor, benign prostatic hyperplasia, transitional zone

## Abstract

Macrophages play a critical role in the process of excessive stromal proliferation of benign prostatic hyperplasia (BPH). In our previous study, we used a BPH mouse model to elucidate a potential mechanism whereby macrophage infiltration promotes stromal cell proliferation in the prostate *via* the androgen receptor (AR)/inflammatory cytokine CCL3-dependent pathway. In our present study, we used the co-culture system of human macrophages and various prostatic zone stromal cells to further demonstrate that infiltrating macrophages promote prostatic stromal cell proliferation through stromal AR-dependent pathways, and we show that the stroma of TZ and PZ respond to macrophages differently because of differences in stromal AR signaling; this could possibly be one of the key pathways for stromal expansion during BPH development and progression. We hypothesize that AR and different downstream inflammatory mediators between TZ and PZ could serve as potential targets for the future design of therapeutic agents for BPH and our results provide significant insights into the search for targeted therapeutic approaches to battle BPH.

## Introduction

Excessive stromal proliferation is an important characteristic for the pathogenesis of benign prostatic hyperplasia (BPH), and macrophages play a critical role in this process (Bianchi-Frias et al., 2010[3]; Dillner et al., 2003[4]; Kindblom et al., 2003[6]). In our previous study, we used a BPH mouse model to elucidate a potential mechanism whereby macrophage infiltration promotes stromal cell proliferation in the prostate *via* the androgen receptor (AR)/inflammatory cytokine CCL3-dependent pathway, and this could be one potential mechanism for stromal expansion in the genesis and development of BPH (Wang et al., 2012[12]). 

Although we have uncovered a significant mechanism in the development of BPH in animal models, there is still a limitation to the study because we did not consider the unique characteristics of the zonal structure of the human prostate, which presents important clinical implications for the occurrence of the disease in the prostate (Abate-Shen and Shen, 2000[1]; Wang et al., 2011[13]; Xia et al., 2001[15]; Love et al., 2009[7]; van der Heul-Nieuwenhuijsen et al., 2006[11]). Interestingly, clinical observation shows that each zone of the prostate has a specific susceptibility to a different disease. BPH manifests a predilection for the transitional zone (TZ), but rarely for the peripheral zone (PZ). Therefore, the animal model of benign prostatic hyperplasia (BPH) cannot completely explain the pathogenesis of prostatic hyperplasia. We therefore hypothesized that macrophages (as well as the AR signaling pathway), may be different between the two different zones, PZ and TZ; and that this may be a potential mechanism for the BPH preference for the transitional zone. 

In the present study, we used the co-culture system of human macrophages and various prostatic zone stromal cells *in vitro* to further study macrophage-mediated proliferation of prostate transitional zone and peripheral zone stromal cells, as well as to verify the differential roles played by the AR signaling pathway in the above process, in order to investigate the pathogenesis of BPH and inflammation-related BPH.

## Materials and Methods

### Primary culture of different prostatic zonal stromal cells from BPH patients

Fresh prostatic specimens from BPH patients were obtained by radical resection of bladder cancer not involving the prostate, at the Shanghai General Hospital (Shanghai, China), and primary culture protocol was followed as described in our previous study (Wang et al., 2012[12], 2011[13]). The experimental protocols were approved by the Shanghai First People's Hospital Medical Ethics Committee. The primary stromal cells (BPHTF/ BPHPF) were identified as described in our previous study cultured with RPMI 1640 supplemented with 10 % fetal bovine serum (FBS), and were maintained at a temperature of 37 °C in a humidified atmosphere of 5 % CO_2_. The stromal cells were used at passages 3-5, and the different prostatic stromal cell subsets derived from the same patient and were used at the same passage for each separate experiment.

### Cell line and co-culture experiments

THP-1 cells were purchased from the American Type Culture Collection (ATCC, Manassas, VA), and cultured in RPMI 1640 with 10 % FBS. Cultures were grown in a humidified atmosphere of 5 % CO_2_ at 37 °C and high humidity. Six-well (0.4 µm) and 24-well (5 µm) transwell plates (Corning Incorporated) were used for co-culture and invasion assay experiments, respectively.

### Plasmids and stable cell lines

For incorporation of the AR-shRNA or scrambled control plasmids into stromal cells, BPHTFs and BPHPFs, lentivirus carrying either control (pLVTHM-scramble) or AR-shRNA (pLVTHM-AR-shRNA) was transfected into HEK293T cells with a mixture of pLVTHM-scramble/pLVTHM-AR-shRNA, psPAX2 (virus packaging plasmid), and pMD2G (envelope plasmid; 4:3:2 ratio) by calcium-phosphate transfection. Culture medium containing virus was collected 32 h after transfection and filtered through a 0.4 µm filter to remove cellular debris or cells. The collected viruses were added to the target cells in the presence of polybrene (2 µg/mL) and incubated for 24 h. Cells were refreshed with culture medium and cultured for another 3 days to let target proteins be expressed. Because the lentiviral vectors express GFP, fluorescence microscopy was used to monitor the infection efficiency *via* evaluation of the green fluorescence signal.

### Cell proliferation assay

Stromal cells were seeded in 24-well plates (10^3^ cells/well) and cultured in RPMI 1640 medium for 24 h. Then, cells were co-cultured with THP-1 cells or control medium and incubated for an additional 2, 4, and 6 days. MTT reagent (Promega, Madison, WI) was added at 2, 4, and 6 days per the manufacturer's instructions. After 4 h of reaction, absorbance of the converted dye was measured at a wavelength of 595 nm-620 nm.

### Cell growth and proliferation assay using a real-time cell analysis (RTCA) system

The growth, proliferation and adhesion kinetics of Vero cells were determined using RTCA technology (ACEA Biosciences, San Diego, CA, USA) and we followed their protocol (AbuBakar et al., 2014[2]; Kho et al., 2015[5]). Briefly, 50 μl of EMEM supplemented with 10 % FBS (cell culture medium) was placed in each well of the E-plate 96 (gold-microelectrode array integrated E-plate; ACEA Biosciences, San Diego, CA USA). E-plate 96 was then connected to the system to obtain background impedance readings. This was to ensure that all wells of E-plate 96 and the connections were in good condition so as to avoid compromising the interpretation of the results. Serial dilutions of 2.0 × 10 ^4^, 1.8 × 10^4^ and 1.5 × 10^4^ cells in 50 μl were prepared, with four replicates for each of the concentrations. These serial dilutions of cell suspensions were then added to the wells containing 50 μl of culture medium. The E-plates were incubated at room temperature for 30 min in a laminar flow cabinet and then placed on the RTCA SP Station located in an incubator at 37 °C for continuous impedance recording. CI values as measured by continuous impedance recordings every 2 min reflected the cellular activities.

### Cell migration assay

*An in-vitro* cell migration assay was performed using 24-well transwell inserts (5 µm) (Corning Incorporated, Shanghai, China) according to the manufacturer's instructions. THP-1 cells (1*10^5^/well) were seeded in the upper chamber of transwell plates, and stromal cells or fresh RPMI 1640 medium and conditioned medium from a stromal cell/macrophage co-culture system (1:1) were placed in the lower chamber. Cells were incubated for 24 h. The migrated cells were counted or assayed using MTT.

### RNA extraction and quantitative real-time PCR analysis

Total RNA was isolated using the Trizol reagent (Invitrogen, CA) according to the manufacturer's instructions. One mg of total RNA was subjected to reverse transcription using Superscript III transcriptase (Invitrogen). RT-PCR was performed according to the manufacturer's manual. Primers used were as follows: 

AR sense, 5′-TATCCTGGTGGAGTTGTG-3′; antisense, 5′-CAGAGTCATCCCTGCTTC-3′; GAPDH sense, 5′- AATGTCACCGTTGTCCAGTTG-3′, antisense, 5′-GTGGCTGG GGCTCTACTTC-3′. 

Quantitative real-time PCR (qRT-PCR) was conducted using a Bio-Rad CFX96 system with SYBR green to determine the levels of mRNA expression of the genes of interest. Expression levels were normalized to the expression of GAPDH RNA. 

### Western blot analysis

Stromal cells were lysed in RIPA buffer (50 mM Tris-HCl, pH 7.4; 1 % NP-40; 150 mM NaCl; 1 mM EDTA; 1 mM PMSF; 1 mM Na_3_VO_4_; 1 mMNaF; 1 mM okadaic acid; and 1 mg/ml aprotinin, leupeptin, and pepstatin). Individual samples (30-35 µg protein) were prepared for electrophoretic separation on an 8-10 % SDS/PAGE gel and then transferred onto PVDF membranes (Minipore, China). After blocking the membranes with 5 % fat-free milk in TBST (50 mM Tris, pH 7.5, containing 0.15 M NaCl, and 0.05 % Tween-20) for 1 h at room temperature, the membranes were incubated with appropriate dilutions of specific primary antibodies overnight at 48 °C. After washing, the blots were incubated with anti-rabbit or anti-mouse IgG HRPs for 1 h. The blots were developed in ECL mixture (Thermo Fisher Scientific Inc., Rockford, IL). 

### Luciferase assay

Stromal cells were plated in 24-well plates and transfected with mouse mammary tumor virus (MMTV)-luc containing ARE sequence using Lipofectamine (Invitrogen) according to the manufacturer's instructions. After transfection, RPMI medium containing charcoal-stripped FBS was added along with various concentrations of dihydrotestosterone (DHT) (0 [ethanol as vehicle control], 1, or 10 nmol/L), and incubated for 48 h. pRL-TK was used as an internal control. Luciferase activity was measured using Dual-Luciferase Assay (Promega, Shanghai, China) according to the manufacturer's manual.

### Immunohistochemistry

Prostatic tissues were fixed in 10 % (v/v) formaldehyde in PBS and embedded in paraffin, and cut into 5-mm tissue sections. Prostate sections were deparaffinized in xylene solution and rehydrated using graded ethanol concentrations. Immunostaining was performed using standard protocols. For systematic counting of macrophages or AR positive cells, six ocular measuring fields within a tissue were randomly chosen under a microscope at 400 magnifications. The mean number of human CD68/AR positive cells in these six areas was determined for each case.

### Statistics

Data are presented as means ± SD. Differences in mean values between two groups were analyzed by two-tailed Student's t test. P ≤ 0.05 was considered to be statistically significant.

## Results

### Increased distribution of macrophage AR within stroma of human BPH transitional zonal tissues compared with peripheral zonal tissues

**Figure 1:**
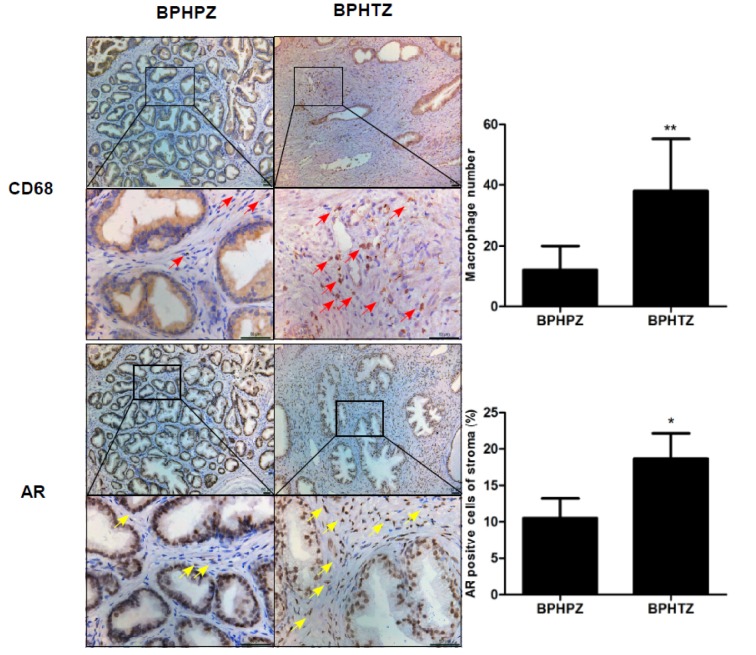
Differential macrophage infiltration and AR distribution between the stroma of prostate transitional and peripheral zones of BPH *in vivo*. Immunohistochemical staining of the transitional zone and peripheral zone of human BPH using anti-CD68 anti-AR antibody (400X). Paraffin-embedded tissue sections of human prostate tissue from the transitional and peripheral zones of human BPH. Red arrow, macrophages; yellow arrow, AR

Our previous study showed that the increased number of infiltrating macrophages in prostatic stromal areas of BPH patients and stromal AR play important roles in macrophage-induced prostatic stromal cell proliferation (Wang et al., 2012[12]). We therefore investigated the different macrophage infiltration and AR distribution patterns between the stroma of prostatic transitional and peripheral zones in human BPH. We first made paraffin-embedded tissue sections of the human prostate transitional zonal tissues and peripheral zonal tissues from BPH patients, and using IHC analysis with anti-CD68 and anti-AR antibodies, we found significantly increased numbers of infiltrating macrophages and density of AR staining in the stroma of the transitional zone (n=10), compared with the peripheral zone (n=10) (Figure 1(Fig. 1)). These results suggest that infiltrating macrophages and AR signaling might play important roles in the development and progression of BPH. In addition, the infiltrating macrophages and AR signaling might play different roles in TZ and PZ, which may explain why BPH occurs almost exclusively in the TZ rather than PZ.

### Stromal cells from the prostatic transitional zone in human BPH may recruit more infiltrating macrophages compared with those from the peripheral zone in a stromal cell/macrophage co-culture system

**Figure 2:**
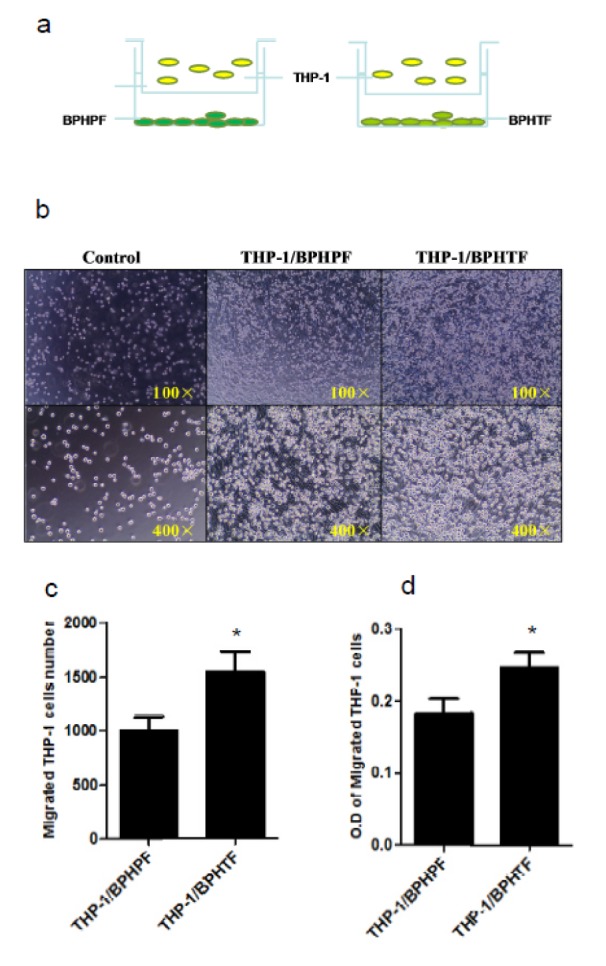
Differences in macrophage infiltration between BPHTF and BPHPF. THP-1 cells (1*10^5^/well) were co-cultured with control medium/BPHTFs or BPHPFs for 24 h in transwell plates (5 µm). Migrated macrophages were counted or assayed with MTT. Results are expressed as means ± standard deviations. Differences between the two groups were analyzed by two-tailed Student's t test; *P < 0.05

To confirm the differential macrophage infiltration that we found *in vivo* between the stroma of prostatic transitional zone and peripheral zone of BPH specimens, we first isolated prostate transitional zonal stromal cells (BPHTF) and peripheral zonal stromal cells (BPHPF) from human BPH patients. Interestingly, we found that BPHTF attracted increased THP-1 infiltration compared with BPHPF during co-culture (Figure 2(Fig. 2)). These data further prove that infiltrating macrophages of prostatic TZ and PZ in BPH might play different roles during prostate BPH progression in BPH, and that the BPHTF show a greater capability in attracting macrophages compared with BPHPF.

### Macrophage-induced stromal cell proliferation of BPHTF is more intense when compared to BPHPF in a stromal cell/macrophage co-culture system

**Figure 3:**
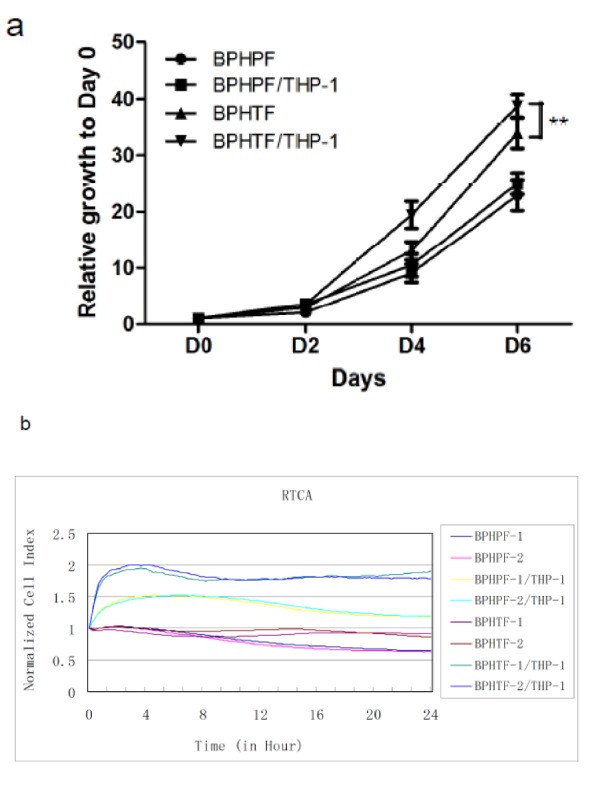
Differences in macrophage-induced stromal cell proliferation between BPHTF and BPHPF. BPHTFs/BPHPFs were co-cultured with THP-1 cells in a transwell and assayed with MTT at 2, 4, and 6 days or RTCA. Differences between the two groups were analyzed by two-tailed Student's t test; *P< 0.05

To investigate the differences in macrophage-induced stromal cell proliferation between BPHTF and BPHPF, we co-cultured BPHTF/BPHPF with THP-1 cells and performed a cell growth assay with 3-(4,5-dimethylthiazol-2-yl)-2,5-diphenyltetrazolium bromide (MTT) and RTCA. We found that THP-1 cells promoted the proliferation of both stromal cell types (BPHTF/BPHPF), and that BPHTF proliferation was more dramatically enhanced by THP-1 cells compared with BPHPF (Figure 3(Fig. 3)). This suggested to us a different role for macrophage-induced prostatic stromal cell proliferation between TZ and PZ, with the BPHTF being more sensitive to macrophage-induced prostatic stromal cell proliferation compared with BPHPF.

### Differential AR signaling between BPHTF and BPHPF

**Figure 4:**
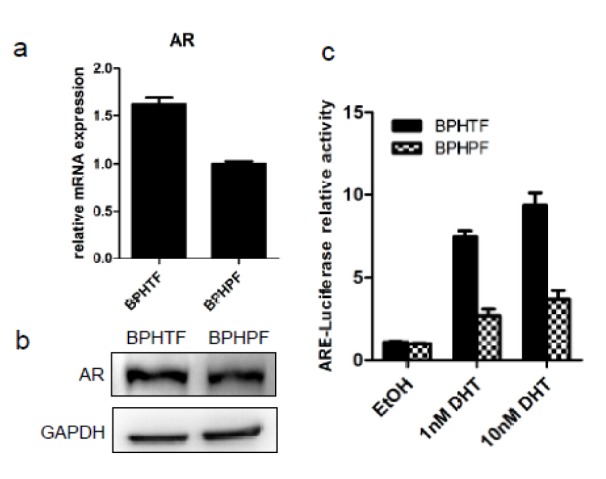
Differences in AR expression and transactivity between BPHTF and BPHPF. (a) qRT-PCR analysis results showed that BPHTF AR mRNA expression was higher compared with that for BPHPF. (b) Western blot analysis results showed that BPHTF AR protein expression levels were higher compared with those for BPHPF. (c) MMTV-luc assay results showed that BPHTF AR transactivity was higher compared to BPHPF.

To identify the differential AR signaling between BPHTF and BPHPF *in vitro* (which may be responsible for macrophage-mediated stromal cell proliferation), we investigated the AR expression by qPCR and found a remarkably higher AR mRNA expression in BPHTFs compared with BPHPFs (Figure 4a(Fig. 4)); this was further confirmed by Western blot analysis of protein expression (Figure 4b(Fig. 4)). AR transactivation was also tested in an ARE-driven luciferase assay, and as expected, the AR-mediated ARE-luciferase activity of BPHTFs was significantly higher than that of BPHPFs (Figure 4c(Fig. 4)), suggesting that both the expression and AR transactivation activity of BPHTFs were augmented *vs.* those of BPHPFs. 

### AR signaling molecules may act as potential mediators for the differences in macrophage infiltration and macrophage-induced stromal cell proliferation between TZ and PZ

**Figure 5:**
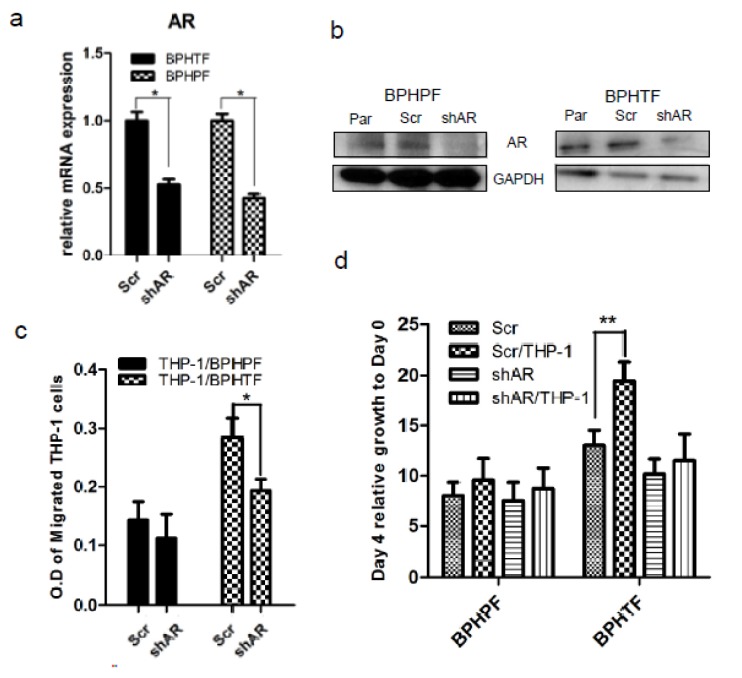
Differential roles for BPHTF/BPHPF AR in macrophage cell infiltration and macrophage-induced stromal cell proliferation. (a) THP-1 (1*10^5^/well) were co-cultured with BPHTF-scr /BPHTF-shAR or BPHPF-scr /BPHPF-shAR cells for 24 h in transwell plates (5 µm). Migrated macrophages were counted or assayed with MTT. (b) BPHTF-scr /BPHTF-shAR or BPHPF-scr /BPHPF-shAR cells were co-cultured with THP-1 cells and assayed with MTT at 2, 4, and 6 days.

We next evaluated whether stromal AR was responsible for the differences in macrophage infiltration and macrophage-mediated stromal cell proliferation during BPH development and progression. We therefore created AR-knockdowns in stromal cells (BPHTF-shAR/BPHPF-shAR) and scrambled control cells (BPHTF-scr/BPHPF-scr). The mRNA and protein levels of BPHTF-shAR/BPHPF-shAR and BPHTF-scr/BPHPF-scr were then examined by quantitative real-time PCR and Western blot assays (Figure 5a-b(Fig. 5)). Intriguingly, we found that knocked-down stromal AR can reverse THP-1 infiltration (Figure 5c(Fig. 5)) and macrophage-induced stromal cell proliferation during co-culture, which indicated that AR signaling in stromal cells is essential for mediating macrophage infiltration and macrophage-induced stromal cell proliferation. In addition, the differences in stromal AR signaling between TZ and PZ may provide a key signal during prostate BPH progression and explain why BPH occurs almost exclusively in the TZ rather than PZ. 

## Discussion

Benign prostatic hyperplasia (BPH) is a frequent disease of middle-aged to elderly men worldwide, and our previous study showed that aberrant proliferation of prostate stromal cells is responsible for BPH pathogenesis (Wang et al., 2012[12]). 

An increasing number of reports link the androgen/AR signals to inflammation with respect to impacting BPH progression. Recently, a study of immune inflammation in 105 BPH specimens revealed that the group with strong immune inflammation had larger prostate volumes, higher AR expression levels and higher serum prostate-specific antigen (PSA) levels (Wu et al., 2012[14]). In our previous study we also investigated the interaction of infiltrating macrophages and stromal cells in BPH, and showed that mouse stromal cells (mPrSC) recruited mouse macrophages (RAW264.7, potentially resulting in the promotion of stromal cell proliferation (Wang et al., 2012[12]). Mechanism dissection found a significant increase in CCL3 expression in both mPrSC cells and RAW264.7 cells, and that neutralizing CCL3 antibody could reduce the migration of RAW264.7 cells toward mPrSC cells and macrophage-enhanced mPrSC cell proliferation (Wang et al., 2012[12]). The *in vivo* Pb-PRL-tg mouse BPH model also confirmed increased macrophage number and CCL3 expression in BPH, and targeting stromal AR *via* deletion of the stromal fibromuscular AR in the Pb-PRL-tg mouse BPH model reduced infiltrating macrophage number and CCL3 expression levels in the prostate. Moreover, immunohistochemical analysis of clinical specimens also showed a higher number of infiltrating macrophages into the stroma and higher expression of CCL3 in human BPH prostates compared with normal prostates. Stromal AR can also promote BPH development *via* enhancement of recruitment of infiltrating macrophages with increased CCL3 expression, resulting in increased stromal cell proliferation (Wang et al., 2012[12]). 

Although we demonstrated an important mechanism of action in the development of BPH, *i.e.*, an inflammatory signaling pathway that may cooperate with the AR signaling pathway to promote the proliferation of prostate stromal cells, and showed a stromal AR→CCL3→ stromal cell expansion signaling pathway in the interpretation of stromal expansion with a potential link between AR and CCL3 in prostatic stroma in an animal model of BPH, our previous study was limited by the fact that there is no other extant study that describes the unique structural characteristics of the human prostate. We understand, of course, that the prostate is divided into different zones and each zone has a specific susceptibility to different diseases. BPH typically arises from the transitional zone, and, in contrast, prostate cancer (PCa) arises from the peripheral zone (van der Heul-Nieuwenhuijsen et al., 2006[11]; McNeal, 1981[8]). During the process of prostate growth, its structure and zonal proportion also change. From adolescence to maturity, the peripheral zone accounts for about 70 %, central zone 5 %, and transitional zone 25 % of the gland, respectively. However, with age and changes in serum hormone levels, the proportion of TZ increases gradually in the elderly (Love et al., 2009[7]). In addition, many studies show that there are different phenotypes, microenvironments, gene expression profiles, and a varied distribution of growth factors (Wang et al., 2011[13]; Xia et al., 2001[15]), suggesting that the prostate transitional and peripheral zones may affect biologic behavioral differences, which cannot simply be explained by anatomical location. The complicated and obvious differences in growth regulatory mechanisms between TZ and PZ are of great importance so as to allow us to further clarify the pathogenesis of BPH.

Although Tang et al. (2007[10]) showed that there was little difference in AR expression between the peripheral zone and TZ hyperplastic nodules, there was a recent study that showed that the periurethral area of the TZ (site of the primary BPH nodule), exhibited the highest levels of both androgens and AR compared with other regions; this suggested that this region may be responsible for the growth-promoting processes inherent to BPH that can result in urinary obstruction (Monti et al., 1998[9]). Our results also showed that macrophage distribution and AR signaling of the prostate transitional zone of BPH patients were higher than those for peripheral zone, resulting in prostatic hyperplasia occurring in the transitional zone.

To determine the potentially different roles for stromal AR in macrophage-induced stromal cell growth between TZ and PZ, we established an *in vitro* co-culture system of macrophages/prostate stromal cells from TZ and PZ of BPH patients; and we assert that our co-culture model can mimic the *in vivo* interaction of macrophages and stromal cells in the inflammatory microenvironment found within the human prostate in BPH. We demonstrated that human prostate stromal cells also attract macrophage infiltration, and that the growth of human prostate stromal cells could also be enhanced by macrophages during co-culture. Interestingly, the stromal cells from TZ may recruit more infiltrating macrophages and show more induced proliferation by macrophages compared with macrophages from PZ. Our use of a stromal cell/macrophage co-culture system supports a potential role for macrophages identified from inflamed stroma of TZ and PZ by immunohistochemical analysis of BPH prostate tissues (Figure 1(Fig. 1)), which also supports the clinical concept of a predilection by BPH for the transitional zone (TZ), and only rarely for the peripheral zone (PZ). Importantly, our findings support differential stromal AR signaling between PZ and TZ, as well as different roles for prostate stromal AR in the interaction of macrophages and stromal cells from TZ and PZ; this shows that stromal AR of TZ more strongly enhance the migration of macrophages, as well as the macrophage-mediated proliferation of prostate stromal cells compared with PZ during co-culture. The present study has established previously unrecognized and different roles for TZ/PZ stromal AR in mediating the cross-talk between macrophages and prostate stromal cells, implicating the possibility that aberrant AR function in prostate stroma may differentially exacerbate inflammatory responses associated with BPH. Similarly, TZ stroma is more sensitive to ablation of AR in the stromal-fibromuscular tissue of prostate to attenuate the enlargement of prostate tissues compared with PZ. This study supports a potential BPH model in which different AR signals synergize with inflammatory signaling pathways so as to promote the proliferation of prostatic stromal cells. 

In conclusion, our findings confirm that AR signaling and its downstream inflammatory mediators TZ and PZ behave differently in enhancing macrophage infiltration, and promote proliferation of prostate stromal cells. There is an urgent need for the identification of new downstream targets for AR or key pathways between TZ and PZ that are essential for preventing the BPH hyperplastic phenotype. We hypothesize that AR and different downstream inflammatory mediators between TZ and PZ could serve as potential targets for the future design of therapeutic agents for BPH.

## Notes

Dongliang Xu and Xingjie Wang contributed equally as first authors to this work. Shujie Xia and Xiaohai Wang (E-mail: wangxiaohai8@163.com) contributed equally as corresponding authors.

## Authors’ contribution

Dong-liang Xu, Shu-jie Xia, Xiao-hai Wang: Project development, Manuscript writing 

Xing-jie Wang, Chenyi Jiang, Yuan Ruan: experimental design and perform, data collection and analysis.

All authors have read and approved the final manuscript.

## Statement of human rights

All procedures performed in studies involving human participants were in accordance with the ethical standards of the institutional and/or national research committee and with the 1964 Helsinki declaration and its later amendments or comparable ethical standards.

## Funding

The project was supported by the National Natural Science Foundation of China (No. 81300625), Shanghai Science and Technology Committee (No. 13DZ1940602), and Shanghai Municipal Commission of Health and Family Planning (No. 20134423).

## Conflicts of interest

The authors declare that they have no conflict of interest.

## Informed consent

Informed consent was obtained from all individual participants included in the study.
